# Identification of prognostic tumor-infiltrating immune cells in endometrial adenocarcinoma

**DOI:** 10.1097/MD.0000000000026170

**Published:** 2021-05-28

**Authors:** Xin-Bin Pan, Yan Lu, De-Sheng Yao

**Affiliations:** a Department of Radiation Oncology; b Department of Gynecologic Oncology, Guangxi Medical University Cancer Hospital, Nanning, Guangxi, PR China.

**Keywords:** CD8 T cell, endometrial adenocarcinoma, macrophage, prognosis

## Abstract

To identify prognostic tumor-infiltrating immune cells of endometrial adenocarcinoma.

The gene expression profiles of endometrial adenocarcinoma were downloaded from the Cancer Genome Atlas (TCGA). The abundance of tumor-infiltrating immune cells in endometrial adenocarcinoma samples was calculated by CIBERSORT algorithm. Kaplan–Meier analysis was used to identify prognostic tumor-infiltrating immune cells.

This study identified 22 kinds of tumor-infiltrating immune cells. Macrophages M0 and CD8 T cells were prognostic factors of endometrial adenocarcinoma. The abundance of macrophages M0 (*P* = .038) was significantly correlated with better prognosis of endometrial adenocarcinoma. In contrast, the abundance of CD8 T cells (*P* = .049) was associated with poor prognosis of endometrial adenocarcinoma.

Tumor-infiltrati macrophages M0 and CD8 T cells were prognostic factors of endometrial adenocarcinoma.

## 1 Introduction

Endometrial cancer is a major cause of morbidity and mortality in women, which accounts for approximately 11000 deaths annually in USA.^[1]^ Endometrial adenocarcinoma is the most common type of endometrial cancer. The 5-year overall survival ranges from 15% to 47% for advanced stage endometrial adenocarcinoma.^[2,3]^ Thus, it is important to identify prognostic factors for endometrial adenocarcinoma.

Tumor-infiltrating immune cells regulate the immune status of tumors. The immune cells involve in the process of tumor progression and metastasis.^[4]^ Exploring tumor-infiltrating immune cells in tumor microenvironment will provide a deeper understanding of immune status of endometrial adenocarcinoma. The tumor-infiltrating immune cells might lead to develop new immunotherapies for patients with poor prognosis. Thus, we conducted this study to identified prognostic tumor-infiltrating immune cells in endometrial adenocarcinoma using CIBERSORT.

## 2 Materials and methods

### 2.1 Data preparation and download

The RNA-seq gene expression profiles and clinical data of patients with endometrial adenocarcinoma was downloaded from The Cancer Genome Atlas (TCGA) dataset (https://cancergenome.nih.gov/). Gene expression data in Fragments Per Kilobase of transcript per Million mapped reads format were then normalized by R software (version 3.6.2). Ethical review and approval were waived for this study, due to all data deriving from TCGA public databases.

### 2.2 Tumor-infiltrating immune cells analysis

Some computational methods, including Microarray microdissection with analysis of differences, linear least-square regression, and digital sorting algorithm, were performed to infer cellular composition of complex gene expression profiles mixtures. However, these approaches are sensitive to high unknown mixture content, experimental noise, and closely related cell types. This limits their utility for tumor-infiltrating immune cells assessment.

CIBERSORT (https://cibersort.stanford.edu/) is an accurate and robust algorithm for calculating the cell composition of a tissue from a gene expression profile.^[5]^ It requires a specialized knowledgebase of gene expression signatures for the deconvolution of cell types of interest. CIBERSORT implements the support vector regression to improve deconvolution performance through a combination of feature selection and robust mathematical optimization techniques. In benchmarking experiments, CIBERSORT was more accurate than Microarray microdissection with analysis of differences, linear least-square regression, or digital sorting algorithm in resolving closely related cell subsets and in mixtures with unknown cell types.

CIBERSORT uses gene expression signatures consistent of ∼500 genes to characterize and quantify each immune cell subtypes. We download CIBERSORTR source code and LM22 gene signature from CIBERSORT web portal (http://cibersort.stanford.edu/) and run it locally. Clinical information and transcriptome data of endometrial adenocarcinoma were combined into a matrix. This study applied the original CIBERSORT gene signature file LM22, which defined 22 kinds of tumor-infiltrating immune cells. The expression profile was normalized and R software was used to run the CIBERSORT algorithm. The number of permutations was set to 1000 to explore the proportion of 22 kinds of tumor-infiltrating immune cells in endometrial adenocarcinoma tissues. The cubic spline algorithm was used to normalize the data.

The bar plot and heat map were drawn to show the composition of 22 kinds of tumor-infiltrating immune cells of each sample. Correlations between 22 kinds of tumor-infiltrating immune cells’ abundance were discussed and a heat map was drawn to display the correlation between cells. CIBERSORT *P* value reflected the statistical significance of the deconvolution results across all immune cell subsets. CIBERSORT *P* value <.05 was used to filter out deconvolution with less significant fitting accuracy.

### 2.3 Identifying survival-related tumor-infiltrating immune cells

Tumor-infiltrating immune cells were divided into high and low groups determined by the median score as a cutoff. Survival analysis was conducted between high and low groups. Survival curves were plotted using Kaplan–Meier analysis. The log-rank test was used to assess differences between survival curves.

### 2.4 Identifying the relationship between tumor-infiltrating immune cells and clinical characters

The relationship between the abundance ratio of tumor-infiltrating immune cells and clinical characters including tumor grade and clinical stage was analyzed by Pearson correlation analysis with corrplot package in R software.

## 3 Results

### 3.1 The tumor-infiltrating immune cells infiltration landscape

This study examined 406 endometrial adenocarcinoma tissues and 19 adjacent nontumor tissues from TCGA. Finally, 152 endometrial adenocarcinoma tissues and 19 adjacent nontumor tissues were included to analysis the distribution and the proportion of tumor-infiltrating immune cells in each sample with CIBERSORT *P* value <.05.

**Figure 1:**
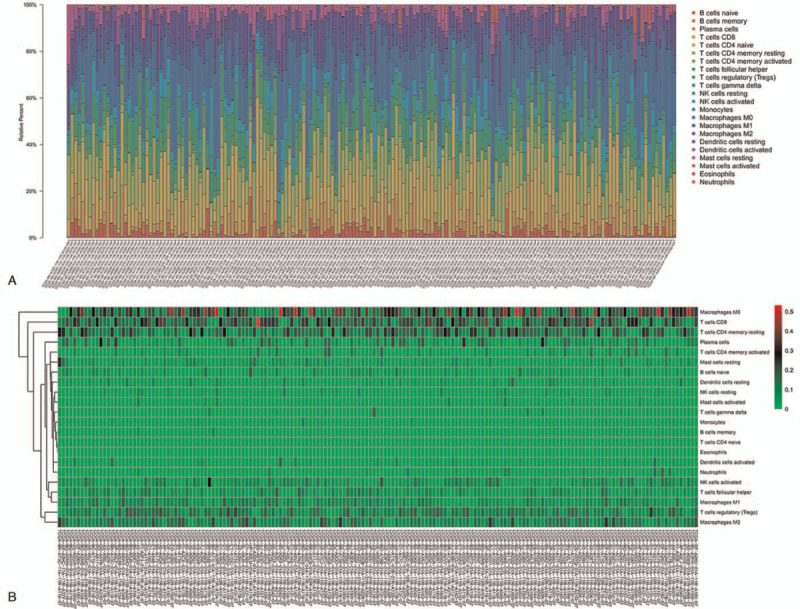
The landscape of immune cells. CIBERSRT algorithm was used to evaluate the microenvironment of the 171 tissues in TCGA cohort. (A) The abundance ratio of immune cells. Each column represents a sample. Each column with a different color and height indicates the abundance ratios of immune cells in this sample. (B) Heat map of the fraction of immune cells.

The proportion of 22 kinds of tumor-infiltrating immune cells was calculated and displayed by plot (Fig. 1). The abundance of tumor-infiltrating immune cells in each sample is different. Macrophages M0 and T cells account for most of the tumor-infiltrating immune cells in endometrial adenocarcinoma microenvironment.

### 3.2 Tumor-infiltrating immune cells between tumor and nontumor tissues

**Figure 2:**
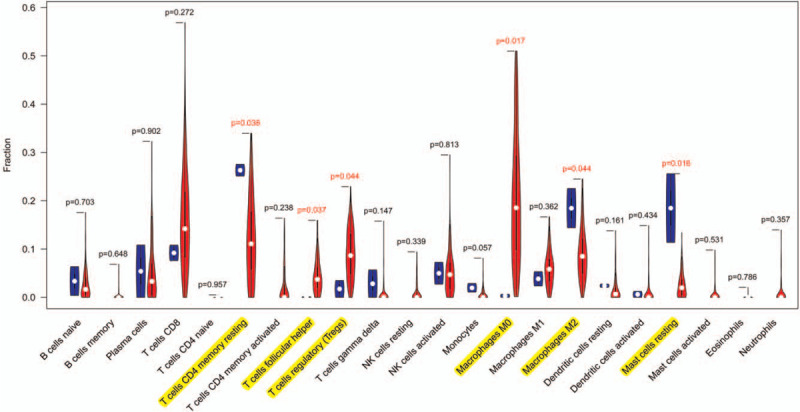
Violin plot for comparison of immune cells fraction difference between tumor and adjacent nontumor tissues. The blue color represents adjacent nontumor tissues. The red color represents tumor tissues. The white points represent median values. Differences between median values are calculated with Wilcoxon rank sum for the significance test. The immune cells that show difference between tumor and adjacent nontumor tissues are marked with yellow.

Figure 2 shows the different fraction of tumor-infiltrating immune cells between tumor and nontumor samples. CIBERSORT immune cell fractions were calculated for samples. The red color represented tumor samples, while the blue color represented adjacent nontumor samples. The white points represented median values. Differences between median values were calculated with Wilcoxon rank sum for the significance test. The immune cells that showed difference between tumor and adjacent nontumor tissues were marked with yellow.

### 3.3 Correlations between tumor-infiltrating immune cells

**Figure 3:**
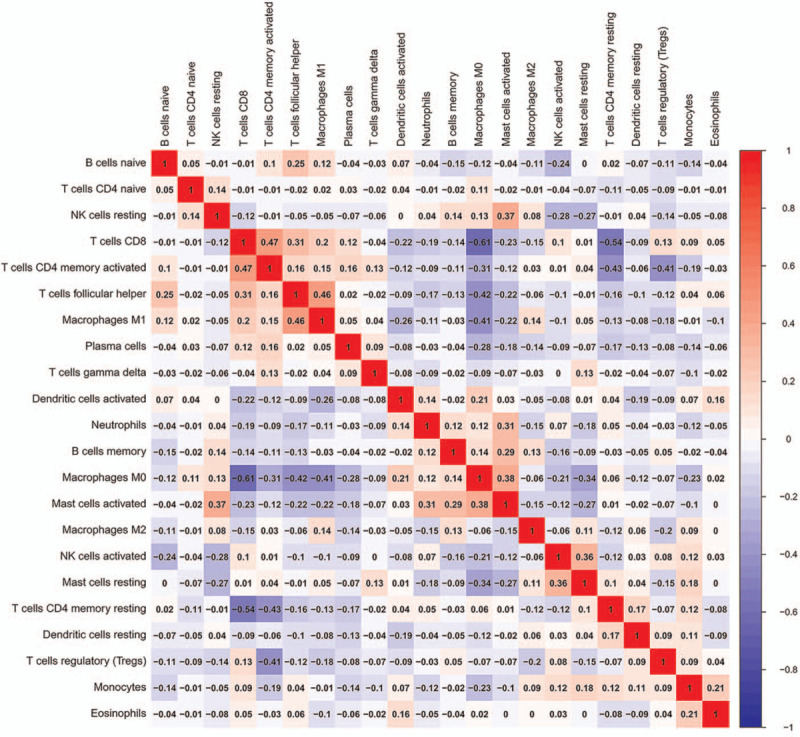
Heatmap showing the correlation between 22 kinds of immune cells. The shade of each tiny color box represents corresponding correlation value between 2 immune cells. The color indicates if they are positive-related (red) or negative-related (blue). For example, T cells CD8 is negatively correlated with macrophages M0 (Correlation coefficient = 0.61) and T cells CD4 memory activated is positively correlated with T cells CD8 (Correlation coefficient = 0.47).

Figure 3 shows the correlation between the 22 kinds of tumor-infiltrating immune cells. The shade of each tiny color box represents corresponding correlation value between 2 immune cells. The color indicates if they are positive-related (red) or negative-related (blue). For example, T cells CD8 is negatively correlated with macrophages M0 (Correlation coefficient = 0.61) and T cells CD4 memory activated is positively correlated with T cells CD8 (Correlation coefficient = 0.47).

### 3.4 Survival-related tumor-infiltrating immune cells

**Figure 4:**
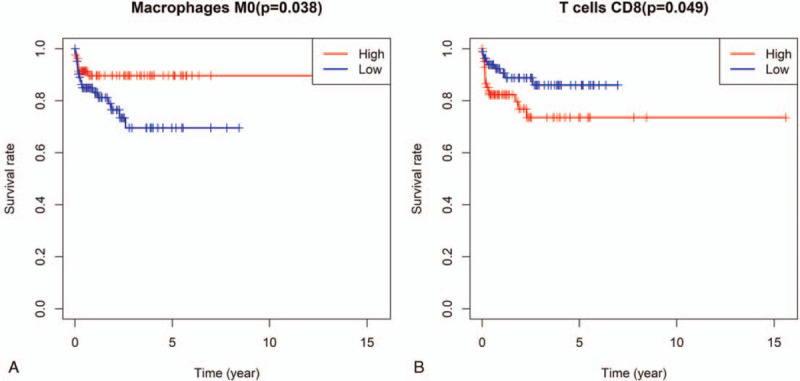
Survival analyses are applied to identify the prognostic value of immune cells. (A) The abundance of macrophages M0 (*P* = .038) is significantly correlated with better prognosis of endometrial adenocarcinoma. (B) The abundance of CD8 T cells (*P* = .049) is associated with poor prognosis of endometrial adenocarcinoma.

The tumor-infiltrating immune cells were divided into high abundance group and low abundance group according to the median values. Survival analysis was carried out (Fig. 4). The abundance of macrophages M0 (*P* = .038) was significantly correlated with better prognosis of endometrial adenocarcinoma. In contrast, the abundance of CD8 T cells (*P* = .049) was associated with poor prognosis of endometrial adenocarcinoma.

### 3.5 Tumor-infiltrating immune cell abundance ratio on clinical characteristics

**Figure 5:**
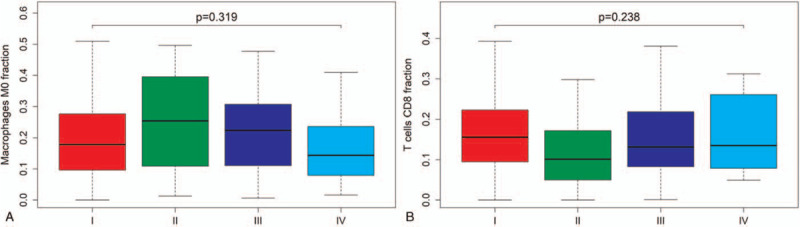
The relationship between clinical stage and overall survival with the fraction of immune cells. (A) The fraction of macrophages M0 (*P* = .319) is not correlated with clinical stage of endometrial adenocarcinoma. (B) The fraction of CD8 T cells (*P* = .238) is not correlated with clinical stage of endometrial adenocarcinoma.

Figure 5 shows the relationship between clinical stage and overall survival with the fraction of immune cells. The fraction of macrophages M0 (*P* = .319) was not correlated with clinical stage of endometrial adenocarcinoma (Fig. 5A). The fraction of CD8 T cells (*P* = .238) was not correlated with clinical stage of endometrial adenocarcinoma (Fig. 5B).

**Figure 6:**
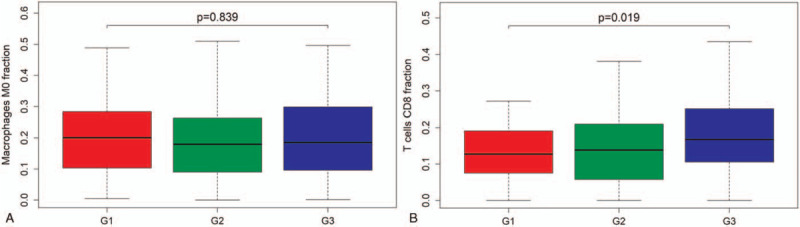
The relationship between tumor grade and overall survival with the fraction of immune cells. (A) The fraction of macrophages M0 (*P* = .839) is not correlated with tumor grade of endometrial adenocarcinoma. (B) The fraction of CD8 T cells (*P* = .019) is significantly correlated with tumor grade of endometrial adenocarcinoma.

Figure 6 shows the relationship between tumor grade and overall survival with the fraction of immune cells. The fraction of macrophages M0 (*P* = .839) was not correlated with tumor grade of endometrial adenocarcinoma (Fig. 6A). In contrast, the fraction of CD8 T cells (*P* = .019) was significantly correlated with tumor grade of endometrial adenocarcinoma (Fig. 6B).

## 4 Discussion

The International Federation of Gynecology and Obstetrics (FIGO) stage is the most important prognostic factor for endometrial adenocarcinoma. Treatments for endometrial adenocarcinoma are mainly based on the FIGO stage. However, the FIGO stage is based on anatomical information, which does not reflect the biological heterogeneity. Patients with the same FIGO stage may have different survivals. Thus, it is important to identify prognostic biomarkers that reflect the biological heterogeneity of endometrial adenocarcinoma. This study revealed that macrophages M0 and CD8 T cells were prognostic factors for endometrial adenocarcinoma. The results provided new prognostic biomarkers for endometrial adenocarcinoma.

The prognostic value of tumor-infiltrating macrophages for cancers is inconsistent.^[6–9]^ It was reported that tumor-infiltrating macrophages was not a prognostic factor for endometrial adenocarcinoma.^[10]^ In contrast, Soeda et al^[11]^ found that patients with high abundance of macrophages had worse progression-free survival (*P* = .0031) and overall survival (*P* = .0085) than those with low level of macrophages. Similarly, our study revealed that tumor-infiltrating macrophages was a prognostic factor.

In gastric cancer, high abundance of tumor-infiltrating macrophages M1 provided a better overall survival (HR = 0.45, 95% CI 0.32–0.65, *P* < .05).^[12]^ In hepatocellular carcinoma, a high density of macrophages M2 was a risk factor for overall survival (HR = 1.58, 95% CI = 1.15–2.00, *P* < .05).^[13]^ In ovarian cancer, elevated ratio of macrophages M1/M2 was a protective factor for overall survival (HR = 0.510, 95% CI: 0.264–0.986, *P* = .045).^[14]^ However, our study revealed that tumor-infiltrating macrophages M1 and M2 were not prognostic factors for overall survival. Only tumor-infiltrating macrophages M0 was a prognostic factor.

CD8 T cells are activated by the macrophages and subsequent destruct tumor cell together. Previous study reported that tumor-infiltrating macrophages had a very strong direct correlation with frequency of tumor cell apoptosis (*P* < .0001) and degree of CD8 T cells (*P* = .0004).^[15]^ Thus, tumor-infiltrating CD8 T cells were reported to be associated with a favorable survivals.^[16]^ However, our study revealed that high abundance of CD8 T cells was a risk factor for overall survival (*P* = .049). Moreover, high abundance of macrophages M0 (*P* = .038) was significantly correlated with better prognosis of endometrial adenocarcinoma. The results of the current study should be treated with caution. Further studies should be performed to verify our results.

Limitation of our study should be considered. Distributions of endometrial adenocarcinoma tissues and adjacent nontumor tissues were different. There seemed to be many extreme cases among adenocarcinoma tissues. The possible interpretations are the following:

1. The results might be related to the purity of tumor tissues included in the TCGA.
2. This study examined 406 endometrial adenocarcinoma tissues and 19 adjacent nontumor tissues from TCGA.

Finally, 152 endometrial adenocarcinoma tissues and 19 adjacent nontumor tissues were included to analysis the distribution and the proportion of tumor-infiltrating immune cells in each sample with CIBERSORT *P* value <.05. The cut-off *P* value using in this study might exclude too many endometrial adenocarcinoma tissues.

In conclusion, tumor-infiltrating macrophages M0 and CD8 T cells were prognostic factors of endometrial adenocarcinoma.

## Author contributions

**Conceptualization:** Xin-Bin Pan, De-Sheng Yao.

**Data curation:** Xin-Bin Pan, Yan Lu.

**Formal analysis:** Xin-Bin Pan.

**Methodology:** Yan Lu.

**Resources:** Yan Lu.

**Software:** Yan Lu.

**Writing – original draft:** Xin-Bin Pan.

**Writing – review & editing:** De-Sheng Yao.
